# Identification of Gene Loci That Overlap Between Mental Disorders and Poor Prognosis of Cancers

**DOI:** 10.3389/fpsyt.2021.678943

**Published:** 2021-06-28

**Authors:** Ji-li Xu, Yong Guo

**Affiliations:** ^1^The First Clinical Medical College, Zhejiang Chinese Medical University, Hangzhou, China; ^2^Department of Medical Oncology, The First Affiliated Hospital of Zhejiang Chinese Medical University, Hangzhou, China

**Keywords:** psychiatric disorder, cancer, risk genes, prevention, target

## Abstract

**Background:** Co-morbid psychiatric disorders are common in patients with cancers, which make the treatment more difficult. Studying the connection between mental disease-related genes and the prognosis of cancers may potentially lead to novel therapeutic methods.

**Method:** All mental disorders genes were selected from published articles. The correlations between the expression of these genes and the prognosis of different cancers were analyzed by starBase v2.0 and TIMER. The molecular functions, reactome pathways, and interactions among diverse genes were explored via the STRING tool.

**Results:** 239 genes were identified for further survival analysis, 5 of which were overlapping genes across at least five cancer types, including RHEBL1, PDE4B, ANKRD55, EPHB2, and GIMAP7. 146 high-expression and 157 low-expression genes were found to be correlated with the unfavorable prognosis of diverse cancer types. Tight links existed among various mental disease genes. Besides, risk genes were mostly related to the dismal outcome of low-grade glioma (LGG) and kidney renal clear cell carcinoma (KIRC) patients. Gene Ontology (GO) and reactome pathway analysis revealed that most genes involved in various critical molecular functions and primarily related to metabolism, signal transduction, and hemostasis.

**Conclusions:** To explore co-expression genes between mental illnesses and cancers may aid in finding preventive strategies and therapeutic methods for high-risk populations and patients with one or more diseases.

## Introduction

Psychiatric disorders are prevalent worldwide and have been a global public health challenge. Approximately 450 million people are suffering from a variety of mental disorders. Although progress has been made in treatment for patients with difficult psychiatric disorders, the current therapeutic regimens remain unsatisfactory. Clinically, bipolar disorder (BD), anxiety disorder, schizophrenia, obsessive-compulsive disorder (OCD), autism, post-traumatic stress disorder (PTSD), and depression are considerably common psychiatric diseases. BD is a complex mental illness with a prevalence of 0.8–1.2% in the population worldwide, characterized by alternating episodes between mania and depression (1, 2). People with BD have a lifelong elevated risk of suicide and a heritability of 60–80% (3, 4). Anxiety disorder, including generalized anxiety disorder (GAD), panic disorder, and social phobia, is highly prevalent and 20–40% heritable (5). Anxiety disorder is common and closely correlated with severe distress, functional impairment, and economic burden (6, 7). Schizophrenia is a common and highly heritable psychiatric disorder and many researchers have tried to explain the association of genetic polymorphisms with schizophrenia. However, numerous genes contribute to the risk of schizophrenia and it is difficult to find the molecular mechanisms of the etiology of schizophrenia. OCD is a potentially disabling disorder and affects ~2–3% of the population (8). ‘A published article has implied that OCD is heritable and childhood onset has a greater genetic influence than adults (9). In addition, the genetic mechanism of OCD is complex with multiple genetic variants leading to its etiology. Autism belongs to a spectrum disorder and affects more boys than girls (10). Autism is often diagnosed in early childhood and the median prevalence of autism is about 0.62% (11, 12). Epidemiological studies have demonstrated that autism has a strong genetic basis (13). Copy number variants (CNV) in the genome of autism patients indicate the role of several genes in the pathogenesis of autism. Besides, autism-related genes can help understand several molecular pathways of autism-associated behaviors. PTSD is a common mental disorder correlated with traumatic events. Genetic vulnerability serves a significant role in the onset of PTSD (14). Thus, gene expression levels are utilized to identify biological pathways related to the disorder. Depression is one of the most prevalent psychiatric disorders with ~350 million patients worldwide (15). Depression adversely affects physical health and psychosocial function and the most severe consequence is suicide. The occurrence of depression results from a combination of various factors including heredity, environment, and endocrine (16). A crucial step to have a better understanding of the pathogenesis of depression is to investigate its underlying genetic determinants. Taken together, psychiatric disorders are influenced by thousands of genetic variants in combination with environmental factors. Meanwhile, cancer is also a complex multi-genic disease caused by somatic mutations in tumor suppressor genes and oncogenes combined with genetic polymorphisms in cancer susceptibility genes. Studies showed that mental illnesses are relevant to increased mortality in people with cancers (17).

Our research aimed to select genes from previously published studies that are related to the onset of diverse psychiatric illnesses. We further explored the influence of these genes on the prognosis of 32 cancer types. To find common causative genes in both mental disorders and cancers may help improve the prevention and therapy. Thus, it is highly valuable to identify co-expressed genes as shared targets for the treatment of patients with both diseases or detection of people at high risk.

## Methods

### Literature Search

In this study, we searched PUBMED to identify eligible original studies and reviews of psychiatric disease-related genes. The selection criteria were as follows: (i) period of the search was limited to articles published after 1 January 2010; (ii) only authoritative journal articles would be included to guarantee the high quality of data; (iii) The sample size must be large and the study provides complete and comprehensive data. The search terms used were “bipolar disorder,” “anxiety disorder,” “schizophrenia,” “obsessive-compulsive disorder,” “OCD,” “autism,” “post-traumatic stress disorder,” “PTSD,” “depression,” “depressive disorder,” “suicide” in combination with “gene,” “genome” or “epigenetics.” No restrictions on language were applied to the literature search. All included studies were published in the last 5 years except for one article on schizophrenia. The authors' names, journal names, and significantly associated genes were all recorded. Then the selected genes were utilized to further perform the prognosis and functional analysis across 32 cancer types.

### Statistical Analyses

This study collected numerous genes related to the onset of mental illnesses from already published articles. The differential expression of selected genes in diverse normal tissues and cancer tissues was analyzed using the Tumor Immune Estimation Resource (TIMER) database (http://timer.comp-genomics.org/) (18) and prognostic values of differently expressed genes in 32 cancer types were performed by starBase v2.0 (http://starbase.sysu.edu.cn/) (19). The molecular functions and reactome pathways of vital genes across diverse cancers as well as the interactions between different genes were explored using STRING (http://string-db.org/) (20). Correlation between genes was calculated using the Pearson correlation coefficient.

*P* < 0.01 were considered as statistically significant.

## Results and Discussion

### Results

Twelve relatively recent and comprehensive articles were identified based on screening criteria (21–32) and psychiatric disorder-related genes were obtained from selected published articles (Table 1). A total of 239 genes were collected, including 44 bipolar disorder-related genes, 13 anxiety-related genes, 8 schizophrenia-related genes, 11 OCD-related genes, 14 autism-related genes, 10 PTSD-related genes, 90 depression-related genes, and 58 suicide-related genes. Of these, 5 genes were found to be overlapping genes across at least 5 cancers including RHEBL1, PDE4B, ANKRD55, EPHB2, and GIMAP7. High expression of 146 genes (Supplementary Table 1a) and low expression of 157 genes (Supplementary Table 1b) were correlated with poor prognosis of different cancers. These genes affect the prognosis of different cancers with different expression levels. Increased expression of BD-related gene RHEBL1 showed strong correlations with the poor prognosis of 5 cancer types. Besides, VRK2, CACNA1C, and DDN were notably linked with worse outcomes of 3 cancers. Decreased expression of TMEM108, ADCY2, ITIH1, and HDAC5 had a deleterious effect on the prognosis of 3 cancers.

**Table 1:** Psychiatric disorder-related genes from 12 published articles.

**References**	**Patient characteristics**	**Disease-related genes**
Li et al. (19)	Bipolar disorder (BD)	TMEM108, VRK2, and RHEBL1
Harrison et al. (22)	Bipolar disorder	LMAN2L, ZNF804A, TRANK1, ADCY2, MIR2113, POU3F2, SYNE1, MAD1L1, ANK3, TENM4, CACNA1C, DDN, and ERBB2
Vieta et al. (23)	Bipolar disorder	PTGFR, LMAN2L, TRANK1, ADCY2, MIR2113, POU3F2, SYNE1, MAD1L1, ELAVL2, ADD3, ANK3, TENM4, CACNA1C, RHEBL1, DHH, DGKH, ERBB2, NCAN, and TRPC4AP
Stahl et al. (24)	Bipolar disorder	PLEKHO1, LMAN2L, SCN2A, ITIH1, CD47, FSTL5, ADCY2, SSBP2, RIMS1, POU3F2, RPS6KA2, THSD7A, SRPK2, MRPS33, ANK3, ADD3, FADS2, PACS1, PC, SHANK2, CACNA1C, STARD9, TRANK1, ZNF592, GRIN2A, HDAC5, ZCCHC2, NCAN, and STK4
Wray et al. (25)	Major depression disorder	RERE, SLC45A1, NEGR1, DENND1B, VRK2, NR4A2, GPD2, TOPAZ1, TCAIM, ZNF445, RSRC1, MLF1, SLC30A9, DCAF4L1, MEF2C, TENM2, FBXL4, TMEM106B, VWDE, PUM3, ASTN2, DENND1A, LHX2, SORCS3, PAUPAR, ELP4, PAX6, SOX5, ENOX1, LACC1, CCDC122, OLFM4, LRFN5, SYNE2, ESR2, DLST, PROX2, RPS6KL1, BAG5, APOPT1, RBFOX1, SHISA9, CPPED1, PMFBP1, DHX38, CRYBA1, MYO18A, NUFIP2, DCC, RAB27B, CCDC68, TCF4, L3MBTL2, and CHADL
Howard et al. (26)	Depression	GRM5, DRD2, ERBB4, GRM8, CACNA1E, HLA-B, KLC1, CELF4, NRG1, KDM3A, ESR2, LRP1B, XRCC3, ANKK1, LST1, TYR, HSPA1A, KMT2A, ESRRG, BAZ2B, EP300, HLA-DQB1, BCHE, FHIT, PSORS1C2, ASIC2, HLA-DQA1, RBMS3, HTT, EPHB2, BAD, TOP1, ATP1A3, PTPRS, KYNU, SERPING1, and CACNA2D1
Meier and Deckert (27)	Anxiety disorder	PKP1, TMEM132D, BDKRB2, CAP2, STXBP6, THBS2, MTCH1, FGD2, RBFOX1, GLRB, MFAP3L, CAMKMT, and PDE4B
Schizophrenia Working Group of the Psychiatric Genomics Consortium (28)	schizophrenia	DRD2, GRM3, GRIN2A, SRR, GRIA1, CACNA1C, CACNB2, and CACNA1I
IOCDF-GC and OCGAS (29)	obsessive-compulsive disorder (OCD)	CASC8, CASC11, GRID2, KIT, ASB13, RSPO4, DLGAP1, PTPRD, GRIK2, FAIM2, and CDH20
Vorstman et al. (30)	Autism	KATNAL2, POGZ, TBR1, ADNP, SYNGAP1, GRIN2B, ANK2, ARID1B, SCN2A, DYRK1A, CHD8, MECP2, NLGN4X, and SYNE1
Daskalakis et al. (31)	Posttraumatic stress disorder (PTSD)	RORA, TLL1, ADCY8, PRTFDC1, TBC1D2, ANKRD55, ZNF626, NLGN1, OR11L1, and KLHL1
Niculescu and Le-Niculescu (32)	Suicidality	LDHB, ARNTL2-AS1, FAH, CTXND1, PGBD5, NARG2, PHLDB2, KCNMB2, ABI3BP, LUZP2, PDE4B, FAM114A2, RBFOX2, PREX1, KIAA1549L, MRAP2, CEP162, ACSL6, LACTB, PRKAG2, AGBL2, GIMAP1, GIMAP7, NUB1, MTNR1A, STAT1, SP140, ABCB8, SLC7A1, FNDC3A, ETV2, ADAM10, RCBTB2, CYP4V2, GIMAP4, HTR2A, GIMAP5, AQP9, ALDH1A2, RHEB, MSRA, CACHD1, CACNA1D, CR1, CRISPLD2, GABRR2, GNAS, GRIN2B, GSN, MAP3K9, PFN2, PRSS3, RALGPS1, RETREG1, RNASEH2B, SYTL3, TSPAN2, and UBE2H

As for anxiety-related genes, high expression of THBS2 and low expression of PDE8B exhibited tight linkages with poor outcomes of 3 and 6 cancers, respectively. Overexpressed CACNA1C and DRD2 both exhibited close connections with 4 cancers among 8 schizophrenia genes. In OCD-related genes, increased expression of CASC8, CASC11, and PTPRD had the most extensive associations with 3 cancers.

Among autism-related genes, patients with low KATNAL2 expression were associated with a worse outcome of 3 cancers. TLL1 and PRTFDC1 were both PTSD-related genes and elevated expression of these two genes were related to the unfavorable prognosis of 4 cancers. Besides, lowly expressed ANKRD55 was linked to the dismal prognosis of 5 cancers. Elevated expression of depression-related genes including VRK2, DRD2, BCHE, and EPHB2 had close relationships with poor survival of at least 4 cancers. Meanwhile, low expression of SLC30A9, LRP1B, FHIT, HTT, ATP1A3, and SERPING1 predicted a worse prognosis of 3 cancers. The associations between suicide genes and cancers were also explored. Various high and low expression of suicide genes has been implicated in worse prognosis of different cancer types, especially ABI3BP, PDE4B, MRAP2, GIMAP1, GIMAP7, and RETREG1.

In addition, among highly expressed genes, most BD-related genes were clustered in Kidney Renal Clear Cell Carcinoma (KIRC), LGG (Brain Lower Grade Glioma), LIHC (Liver Hepatocellular Carcinoma), and MESO (Mesothelioma). The majority of depression-related genes centered on ACC (Adrenocortical Carcinoma), KIRC, LGG, Uterine Corpus Endometrial Carcinoma (UCEC), and UVM (Uveal Melanoma). Besides, suicide-related genes were mostly associated with KIRC, LGG, and UVM. As for lowly expressed genes, BD, depression, and suicide-related genes were all mainly correlated with KIRC and LGG. Other, most depression and suicide- related genes also exhibited close links to SKCM (Skin Cutaneous Melanoma). Furthermore, a variety of OCD-related genes were observed to be related to LGG.

In addition, the relationships between genes in each mental illness were further revealed (Supplementary Tables 2a,b). Among the high expression genes, NCAN and LMAN2L have a strong correlation, with a score of 0.725 among BD-related genes. Additionally, TENM4, and TRANK1 both displayed relatively close associations with CACNA1C (cor > 0.6). Besides, CACNA1C was a shared gene between BD and schizophrenia. Schizophrenia genes CACNB2 and CACNA1C showed a robust correlation (cor = 0.997). Tight linkages were observed between autism-related genes POGZ and KATNAL2 (cor = 0.793). Many depression-related genes displayed numerous strong interactions. NRG1 and ERBB4, HLA-DQB1 and HLA-DQA1, LILRB1 and HLA-B, GRM8 and DRD2, HLA-DQB1 and HLA-B, HLA-DQA1 and HLA-B, DRD2 and ANKK1, KLC1 and HLA-DQA1, KLC1 and HLA-DQB1 all showed strong proximity (cor ≥ 0.9). As for suicide-related genes, only a small portion of the genes indicated relatively high relevance. The highest association score was 0.932 between GIMAP5 and GIMAP4. No significant relevance was detected among anxiety disorder and PTSD-related genes. Similar results could be obtained in the lowly expressed group except for several slight differences. Close associations between different psychiatric disease-related genes have also been analyzed (Supplementary Table 3). Depression gene CACNA2D1 was significantly correlated with schizophrenia genes CACNB2 and CACNA1C with scores of 0.995 and 0.983, respectively. The correlation coefficient between OCD-related gene DLGAP1 and autism-related gene NLGN4X was 0.953. It seemed that close associations among diverse genes were not limited to a single disease.

Besides, the relationships between different psychiatric disorder-related genes that influenced the prognosis of the same cancer type were also studied. We performed the analysis both in the high expression group (Supplementary Table 4a) and low expression group (Supplementary Table 4b), mainly concentrating on ACC, KIRC, LGG, and UVM. Besides, the majority of mental disorder-related genes were correlated with LGG and KIRC. Molecular function and reactome analysis was performed on several crucial genes across at least 3 cancers (Supplementary Tables 5a,b). In terms of molecular function, most of the crucial genes were involved in either “kinase activity” or “binding.” And reactome pathway analysis showed that the majority of genes were associated with “metabolism,” “signal transduction,” and “hemostasis.” The molecular function and pathway analysis may contribute to understanding the common pathogenesis between psychiatric disorders and different cancers.

### Discussion

Psychiatric disorders have increased in prevalence worldwide, which cause high mortality and reduced quality of life. Studies have indicated that mental disorders can be associated with increased mortality in patients with cancer (17). However, the clear explanation of the correlation between these two diseases remains unknown. Our study aims to unravel the relationship at the molecular level. And this is the first study on co-expression genes in psychiatric disorders and various types of cancers. Our study was intended to provide preventive and therapeutic strategies for four populations. The first one is that genes are both highly or lowly expressed in psychiatric diseases and cancers. These genes may serve as potential therapeutic targets in both disorders. Meanwhile, in our view, these genes can be assessed in humans to validate the influence of these genes on cancers or psychiatric disorders. The second one is that the same gene is expressed differently in two diseases. In this condition, targeted therapy is not applicable. Thus, it is important to identify and screen genes that are both high or low expression in mental illnesses and cancers. Taking an example, the CACNA1C gene was widely expressed in the entire nervous system. Previous studies have demonstrated a significant involvement of CACNA1C in sleep-wake regulation and reduced CACNA1C function would increase somnolence or sleep debt (33). Sleep deprivation can lead to mood episode among patients with BD. Deletion of CACNA1C during embryonic development resulted in altered adult behavior related to schizophrenia. Moreover, low expression of CACNA1C was significantly implicated in LGG patients. PDE4B, as a common risk gene between anxiety disorder and suicidality, has been reported to promote angiogenesis and tumor growth (34). A previous study has found that inhibiting PDE4B could reduce anxiety and improve cognition ability (35). Our research indicated that increased expression of PDE4B was significantly correlated with the dismal prognosis of Thymoma (THYM) patients. More detailed function of PDE4B in cancers and mental illnesses requires further analyses. DRD2 was regarded to be responsible for schizophrenia and all antipsychotics were thought to have ameliorating effects on positive symptoms through blocking the level of DRD2 (36). High expression of DRD2 was linked to poor prognosis of Uterine Corpus Endometrial Carcinoma (UCEC) and Uveal Melanoma (UVM). Therefore, DRD2 might be a common target for the treatment of these diseases. Despite the advancements have been made, many genes remain to be identified. Expression levels of these psychiatric disorder-related genes could be detected by RT-qPCR that we can evaluate the results in Supplementary Tables 1a,b. To find genes with the same expression levels in two or more diseases would help us identify the high-risk population and find new therapeutic methods. In addition, we should pay more attention to the co-expression genes between cancers and depression as well as suicidal behavior. Patients with abnormal expression of these genes usually have a higher risk of suicide.

Psychiatric disorders were known to be multifactorial diseases by the contribution of diverse susceptibility genes. The risk of each mental disease was influenced by the interaction among different susceptibility genes. Close connections between different genes of the same psychiatric disorder have been identified. These genes should be more especially vigilant in preventing the onset of mental disorders. In addition, tight correlations between different psychiatric disease-related genes also have been recognized. As an example, the schizophrenia gene CACNB2 had a close association with the depression gene CACNA2D1 with a score of 0.995. Studies have indicated that comorbidity rates were high among individuals with mental disorders. Anxiety disorder was highly comorbid with schizophrenia as well as other psychiatric illness, such as BD (37). Approximately two-thirds of PTSD patients had at least two other mental illnesses, such as depression or BD. Thus, regarding the high comorbidity of psychiatric diseases, we should give more attention to the co-expression genes among different mental disorders. Furthermore, these risk genes are still associated with several cancer types. They may be utilized as targets for screening and treatment between cancers and mental disorders.

Our research also displayed the distribution of all psychiatric illnesses genes in each cancer type. We found that no matter high or low expression, most genes were concentrated in KIRC and LGG. Additionally, highly expressed genes were also related to ACC and UVM. Numerous genes with tight linkages in these 4 cancers were listed. The interaction between these gene combinations may be involved in cancer pathogenesis. Especially among mental disorders, LGG and KIRC, risk genes frequently coincided. Patients who are affected by one disease and carry disease-causing genes should pay more attention to their health status. Patients with psychiatric disorders may be more likely to have LGG or KIRC. However, the specific mechanisms among these diseases remain to be fully elucidated. However, the results can provide a potential target therapy against cancers.

Psychiatric disorder-related genes across at least 4 cancer types were further analyzed using the Reactome to predict the pathways in which they were involved. We found that most genes clustered into 3 main groups including signal transduction, metabolism, and hemostasis pathways. The integration of metabolic pathways with multiple signal transduction pathways plays a vital role in many disorders (38). Thus, these two major pathways in the pathogenesis of cancers and psychiatric disorders should receive more attention. Besides, cancer morbidity is a result of non-balanced hemostasis between cell proliferation and cell death in multicellular organisms (39). Except for regulating hemostasis and coagulation, platelets are also crucial regulators of tumor progression (40). The hemostasis pathway may also be an important target for the treatment of mental diseases and cancers. Furthermore, kinase activity and binding were the most frequent molecular functions among different mental illness and cancer patients. Kinases play a significant role in many biological pathways and abnormal kinase activity has been implicated in various diseases. Moreover, unusual protein binding models can lead to cancer invasion and metastasis.

### Limitations

The main limitation of this study was the limited number of mental disease-related genes and our research was not comprehensive enough. Second, the sample size of some cancers in the TCGA database was relatively small, which may reduce the accuracy of our conclusion. The third one was that although our findings showed the relationships between psychiatric disorder-related genes and corresponding cancers, a more in-depth explanation of these associations was not described.

## Data Availability Statement

Publicly available datasets were analyzed in this study. This data can be found at: The data that support the findings of this study are openly available in PUBMED and TCGA database.

## Author Contributions

J-lX: conceptualization, formal analysis, investigation, and writing-original draft preparation. YG: conceptualization, methodology, writing-review, editing, and supervision. All authors contributed to the article and approved the submitted version.

## Conflict of Interest

The authors declare that the research was conducted in the absence of any commercial or financial relationships that could be construed as a potential conflict of interest.
