# Supplementary material for: Identification of Gene Loci That Overlap Between Mental Disorders and Poor Prognosis of Cancers
Source: Front Psychiatry. 2021 Jun 28;12:678943. doi: 10.3389/fpsyt.2021.678943 (PMC8273260; doi:10.3389/fpsyt.2021.678943)
Supplement: Supplementary file 1 [file Table_1.DOCX]

**Supplementary Tables**

**Supplementary Table 1a Poor prognosis role of high expression of psychiatric disorder-related genes in 32 cancer types**

| **Cancer type** | **Bipolar disorder-related genes** | **Anxiety disorder-related genes** | **Schizophrenia-related genes** | **obsessive-compulsive disorder-related genes** | **Autism-related genes** | **Post Traumatic Stress Disorder-related genes** |
| --- | --- | --- | --- | --- | --- | --- |
| **ACC** | RHEBL1, MAD1L1, DDN | - | CACNB2 | CASC8, CASC11 | POGZ, TBR1 | PRTFDC1 |
| **BLCA** | ADCY2, PTGFR, FADS2 | THBS2 | - | PTPRD, FAIM2 | - | TLL1 |
| **BRCA** | - | - | - | - | - | - |
| **CESC** | - | - | - | - | - | TLL1 |
| **CHOL** | - | - | - | - | - | - |
| **COAD** | - | - | - | - | - | - |
| **DLBC** | - | - | - | - | - | - |
| **ESCA** | - | - | - | - | - | - |
| **HNSC** | - | - | - | CASC8 | - | TBC1D2 |
| **KICH** | SRPK2 | - | - | - | - | PRTFDC1 |
| **KIRC** | POU3F2, MAD1L1, NCAN, TRPC4AP , PLEKHO1, FSTL5 | RBFOX1 | - | CASC11, RSPO4, GRIK2, FAIM2 | TBR1 | - |
| **KIRP** | RHEBL1, CACNA1C, DDN, FADS2, STK4 | - | CACNA1C | - | - | - |
| **LAML** | - | - | - | - | - | - |
| **LGG** | VRK2, RHEBL1, TRANK1, ERBB2, DHH, PLEKHO1, ITIH1, SSBP2, STARD9 | BDKRB2,CAP2,  CAMKMT | - | - | KATNAL2 | - |
| **LIHC** | VRK2, RHEBL1, LMAN2L, POU3F2, TRPC4AP , HDAC5, STK4 | MTCH1 | - | - | - | - |
| **LUAD** | - | - | - | - | - | - |
| **LUSC** | - | - | - | - | ANK2 | - |
| **MESO** | RHEBL1, DDN, PTGFR, DHH, SRPK2, FADS2, PACS1 | THBS2, GLRB | - | - | - | TLL1, PRTFDC1 |
| **OV** | CACNA1C, RPS6KA2, PC | - | CACNA1C | - | - | - |
| **PAAD** | - | - | - | CASC8, CASC11 | - | - |
| **PCPG** | - | - | - | - | - | - |
| **PRAD** | - | - | - | - | - | - |
| **READ** | - | - | - | - | - | - |
| **SARC** | PC | - | - | - | POGZ | - |
| **SKCM** | DDN | PKP1 | - | KIT | - | - |
| **STAD** | - | - | - | PTPRD | - | - |
| **TGCT** | - | - | - | - | - | - |
| **THCA** | - | MFAP3L | - | - | - | - |
| **THYM** | TMEM108 | PDE4B | - | - | - | - |
| **UCEC** | PC | - | DRD2 | GRID2, RSPO4 | MECP2 | NLGN1 |
| **UCS** | - | - | - | - | - | - |
| **UVM** | TMEM108, ADCY2, TENM4 | THBS2, FGD2,  CAMKMT | DRD2 | KIT, PTPRD | KATNAL2 | TLL1, PRTFDC1 |

**Continued**

| **Cancer type** | **Depression-related genes** | **Suicide-related genes** |
| --- | --- | --- |
| **ACC** | RERE, NR4A2, MLF1, PUM3, LHX2, L3MBTL2, XRCC3, PTPRS | STAT1, SLC7A1 |
| **BLCA** | NEGR1, TENM2, PAX6, TCF4, BCHE, RBMS3, CACNA2D1 | GSN, TSPAN2 |
| **BRCA** | - | - |
| **CESC** | - | - |
| **CHOL** | - | - |
| **COAD** | HSPA1A | - |
| **DLBC** | - | - |
| **ESCA** | - | - |
| **HNSC** | BCHE, TOP1 | LDHB, FAH, PFN2 |
| **KICH** | - | LACTB |
| **KIRC** | DCAF4L1, LHX2, RPS6KL1, APOPT1, RBFOX1, PMFBP1, CRYBA1, KLC1, XRCC3, PSORS1C2, EPHB2, BAD, ATP1A3 | AGBL2, SP140, ABCB8, AQP9, GABRR2, GNAS, RNASEH2B |
| **KIRP** | NRG1, ATP1A3 | SLC7A1, RHEB, CACHD1 |
| **LAML** | LRP1B, HSPA1A | - |
| **LGG** | VRK2, DCAF4L1, VWDE, PAUPAR, CCDC122, CPPED1, PMFBP1, HLA-B, ANKK1, LST1, HLA-DQB1, PSORS1C2, HLA-DQA1, EPHB2, KYNU, SERPING1 | FAH, PREX1, LACTB, AGBL2, NUB1, STAT1, SP140, ABCB8, ETV2, CYP4V2, GIMAP4, GABRR2, GSN, SYTL3 |
| **LIHC** | VRK2, ELP4, APOPT1, SHISA9, GRM8, CACNA1E, XRCC3 | CEP162, RHEB, MAP3K9, PFN2 |
| **LUAD** | RSRC1, RAB27B, EPHB2 | LDHB |
| **LUSC** | - | CRISPLD2 |
| **MESO** | TENM2, ELP4, PAX6, NRG1 | GNAS |
| **OV** | MYO18A, ATP1A3 | - |
| **PAAD** | RAB27B, CCDC68, TOP1 | - |
| **PCPG** | RPS6KL1 | - |
| **PRAD** | - | - |
| **READ** | - | - |
| **SARC** | MLF1 | SLC7A1, PFN2 |
| **SKCM** | TENM2 | SLC7A1, ETV2 |
| **STAD** | LRFN5, ERBB4, BCHE | CTXND1, CRISPLD2 |
| **TGCT** | - | - |
| **THCA** | - | - |
| **THYM** | LACC1, HLA-B | PDE4B, PREX1, LACTB, SP140, GIMAP4, GIMAP5 |
| **UCEC** | RSRC1, MLF1, VWDE, ENOX1, SHISA9, DRD2, CACNA1E, CELF4, BCHE, EPHB2 | KIAA1549L |
| **UCS** | ATP1A3 | - |
| **UVM** | SLC45A1, PUM3, ASTN2, DLST, DRD2, GRM8, HLA-B, HLA-DQB1, HLA-DQA1, EPHB2 | PREX1, ACSL6, GIMAP1, SP140, ABCB8, GIMAP4, ALDH1A2, CRISPLD2, GNAS,  RNASEH2B |

**ACC:** Adrenocortical Carcinoma; BLCA: Bladder Urothelial Carcinoma; BRCA: Breast Invasive Carcinoma; CESC: Cervical Squamous Cell Carcinoma and Endocervical Adenocarcinoma; CHOL: Cholangiocarcinoma; COAD: Colon Adenocarcinoma; DLBC: Lymphoid Neoplasm Diffuse Large B-cell Lymphoma; ESCA: Esophageal Carcinoma; HNSC: Head and Neck Squamous Cell Carcinoma; KICH: Kidney Chromophobe; KIRC: Kidney Renal Clear Cell Carcinoma; KIRP: Kidney Renal Papillary Cell Carcinoma; LAML: Acute Myeloid Leukemia; LGG: Brain Lower Grade Glioma; LIHC: Liver Hepatocellular Carcinoma; LUAD: Lung Adenocarcinoma; LUSC: Lung Squamous Cell Carcinoma; MESO: Mesothelioma; OV: Ovarian Serous Cystadenocarcinoma; PAAD: Pancreatic Adenocarcinoma; PCPG: Pheochromocytoma and Paraganglioma; PRAD: Prostate Adenocarcinoma; READ: Rectum Adenocarcinoma; SARC: Sarcoma; SKCM: Skin Cutaneous Melanoma; STAD: Stomach Adenocarcinoma; TGCT: Testicular Germ Cell Tumors; THCA: Thyroid Carcinoma; THYM: Thymoma; UCEC: Uterine Corpus Endometrial Carcinoma; UCS: Uterine Carcinosarcoma; UVM: Uveal Melanoma

**Supplementary Table 1b Poor prognosis role of low expression of psychiatric disorder-related genes in 32 cancer types**

| **Cancer type** | **Bipolar disorder-related genes** | **Anxiety disorder-related genes** | **Schizophrenia-related genes** | **obsessive-compulsive disorder-related genes** | **Autism-related genes** | **Post Traumatic Stress Disorder-related genes** |
| --- | --- | --- | --- | --- | --- | --- |
| **ACC** | TMEM108, ADCY2, TENM4 | - | - | - | - | - |
| **BLCA** | - | - | - | - | - | - |
| **BRCA** | ITIH1 | - | - | - | - | ANKRD55 |
| **CESC** | ITIH1, HDAC5 | FGD2 | - | - | - | ANKRD55 |
| **CHOL** | - | - | - | - | - | - |
| **COAD** | - | - | - | - | - | - |
| **DLBC** | - | - | - |  | KATNAL2 | - |
| **ESCA** | - | - | - | - | - | - |
| **HNSC** | STARD9 | FGD2 | CACNB2 | - | SYNGAP1 | ANKRD55 |
| **KICH** | PACS1, HDAC5 | - | - | - | - | - |
| **KIRC** | ADCY2, SYNE1, ANK3, ERBB2, ADD3, DHH, DGKH, THSD7A, SHANK2, ZNF592, HDAC5 | TMEM132D, CAP2,  MTCH1,  MFAP3L | - | KIT | ARID1B, CHD8, MECP2, NLGN4X, SYNE1 | RORA, TLL1 |
| **KIRP** | ADCY2 | TMEM132D | - | ASB13 | KATNAL2 | - |
| **LAML** | - | - | - | - | NLGN4X | - |
| **LGG** | TMEM108, ZNF804A, ADCY2, CACNA1C, PTGFR, ELAVL2, ADD3, FSTL5, FADS2, PC, SHANK2 | TMEM132D, STXBP6,  MTCH1,  GLRB | GRIA1,  CACNA1C | CASC11, GRID2, ASB13, DLGAP1, PTPRD, GRIK2, FAIM2, CDH20 | GRIN2B | TLL1, PRTFDC1, NLGN1 |
| **LIHC** | ITIH1 | - | - | - | - | - |
| **LUAD** | TMEM108, SCN2A | FGD2, RBFOX1,  PDE4B | - | KIT | SCN2A | RORA, ANKRD55 |
| **LUSC** | - | CAMKMT | - | - | - | - |
| **MESO** | TRANK1 | - | - | - | - | - |
| **OV** | - | - | - | - | - | - |
| **PAAD** | TMEM108, ELAVL2 | - | CACNB2 | - | KATNAL2 | - |
| **PCPG** | LMAN2L | - | - | GRID2 | TBR1 | - |
| **PRAD** | - | - | - | - | - | - |
| **READ** | - | - | - | - | - | - |
| **SARC** | DHH | - | - | - | - | - |
| **SKCM** | VRK2, TRANK1, CD47 | FGD2, PDE4B | CACNA1I | - | - | ANKRD55 |
| **STAD** | - | - | - | - | - | - |
| **TGCT** | - | - | - | - | - | - |
| **THCA** | CD47 | - | - | - | - | - |
| **THYM** | - | - | - | - | - | - |
| **UCEC** | - | - | SRR | CASC8 | - | - |
| **UCS** | - | PKP1 | - | - | - | - |
| **UVM** | SSBP2 | - | - | - | - | - |

**Continued**

| **Cancer type** | Depression-related genes | Suicide-related genes |
| --- | --- | --- |
| **ACC** | SORCS3, RAB27B, FHIT | ABI3BP, LACTB, MSRA |
| **BLCA** | TCAIM, DCAF4L1, DCC | AGBL2 |
| **BRCA** | CACNA1E, LRP1B, FHIT, HLA-DQA1 | MRAP2 |
| **CESC** | L3MBTL2, XRCC3 | SP140, MSRA, SYTL3 |
| **CHOL** | - | - |
| **COAD** | - | - |
| **DLBC** | ATP1A3 | ABCB8 |
| **ESCA** | - | RBFOX2 |
| **HNSC** | HTT | GIMAP1, GIMAP7, GIMAP5 |
| **KICH** | - | - |
| **KIRC** | RERE, GPD2, TCAIM, SLC30A9, TMEM106B, ASTN2, DENND1A, ELP4, LACC1, SYNE2, DLST, BAG5, SHISA9, CPPED1, TCF4, GRM5, NRG1, KMT2A, ESRRG, EP300, HTT | PHLDB2, ABI3BP, FAM114A2, RBFOX2, PREX1, GIMAP1, FNDC3A, RCBTB2, CYP4V2, MSRA, CACHD1, CACNA1D, GSN,  RETREG1 |
| **KIRP** | SLC30A9, BAG5, HTT | CACNA1D, RETREG1 |
| **LAML** | - | FNDC3A, RNASEH2B |
| **LGG** | NEGR1, ASTN2, SORCS3, ENOX1, OLFM4, LRFN5, RPS6KL1, RAB27B, CACNA1E, CELF4, LRP1B, KMT2A, BCHE, ATP1A3 | LDHB, ABI3BP, LUZP2, KIAA1549L, ACSL6,  PRKAG2, HTR2A, SLC7A1, GRIN2B, PFN2,  RALGPS1 |
| **LIHC** | - | GIMAP1, GIMAP7, GIMAP5 |
| **LUAD** | RPS6KL1, RBFOX1, HLA-DQB1 | ABI3BP, PDE4B, PREX1, GIMAP7, RCBTB2,  GIMAP4 |
| **LUSC** | - | - |
| **MESO** | SLC30A9, FHIT, SERPING1 | NUB1, AQP9, CACHD1, RETREG1 |
| **OV** | HLA-B | STAT1 |
| **PAAD** | CCDC122, LRP1B, ATP1A3, PTPRS | CEP162, ACSL6, CACNA1D |
| **PCPG** | L3MBTL2 | MRAP2 |
| **PRAD** | - | - |
| **READ** | ZNF445 | - |
| **SARC** | SERPING1 | GIMAP7 |
| **SKCM** | DENND1B, VRK2, NR4A2, HLA-B, HLA-DQB1, HLA-DQA1, KYNU, SERPING1 | PDE4B, MRAP2, ACSL6, LACTB, GIMAP1, GIMAP7, NUB1, STAT1, SP140, GIMAP4, GIMAP5, CR1, RETREG1, SYTL3 |
| **STAD** | - | - |
| **TGCT** | - | - |
| **THCA** | - | - |
| **THYM** | ZNF445, DHX38, XRCC3 | ADAM10, SYTL3 |
| **UCEC** | - | MRAP2, CYP4V2, GIMAP4 |
| **UCS** | - | - |
| **UVM** | BCHE | PDE4B, KIAA1549L |

**Supplementary Table 2a Correlation coefficient between high expression of psychiatric disorder-related genes**

| **Psychiatric disorder** | **Gene 1** | **Gene 2** | **Correlation score** |
| --- | --- | --- | --- |
| **Bipolar disorder** | NCAN | LMAN2L | 0.725 |
|  | TENM4 | CACNA1C | 0.688 |
|  | TRANK1 | TENM4 | 0.675 |
| **Schizophrenia** | CACNB2 | CACNA1C | 0.997 |
| **OCD** | GRIK2 | GRID2 | 0.631 |
| **Autism** | POGZ | KATNAL2 | 0.793 |
| **Depression** | NRG1 | ERBB4 | 0.998 |
|  | HLA-DQB1 | HLA-DQA1 | 0.998 |
|  | LILRB1 | HLA-B | 0.968 |
|  | GRM8 | DRD2 | 0.940 |
|  | HLA-DQB1 | HLA-B | 0.935 |
|  | HLA-DQA1 | HLA-B | 0.929 |
|  | DRD2 | ANKK1 | 0.903 |
|  | KLC1 | HLA-DQA1 | 0.900 |
|  | KLC1 | HLA-DQB1 | 0.900 |
|  | CACNA2D1 | CACNA1E | 0.893 |
|  | LACC1 | CCDC122 | 0.846 |
|  | NR4A2 | DRD2 | 0.695 |
|  | RBFOX1 | CELF4 | 0.673 |
|  | PAX6 | LHX2 | 0.670 |
|  | PAX6 | ELP4 | 0.633 |
|  | NR4A2 | LHX2 | 0.615 |
| **Suicide** | GIMAP5 | GIMAP4 | 0.932 |
|  | PFN2 | GSN | 0.794 |
|  | GIMAP1 | GIMAP4 | 0.792 |
|  | GIMAP5 | GIMAP1 | 0.646 |

**Supplementary Table 2b Correlation coefficient between low expression of psychiatric disorder-related genes**

| **Psychiatric disorder** | **Gene 1** | **Gene 2** | **Correlation score** |
| --- | --- | --- | --- |
| **Bipolar disorder** | SCN2A | ANK3 | 0.978 |
|  | CACNA1C | ANK3 | 0.755 |
|  | ZNF804A | CACNA1C | 0.709 |
|  | SYNE1 | ANK3 | 0.688 |
|  | ZNF804A | VRK2 | 0.687 |
|  | ZNF804A | ANK3 | 0.685 |
|  | DGKH | ANK3 | 0.669 |
| **Schizophrenia** | CACNB2 | CACNA1C | 0.997 |
|  | CACNB2 | CACNA1I | 0.682 |
| **OCD** | GRIK2 | GRID2 | 0.631 |
| Autism | SYNGAP1 | GRIN2B | 0.805 |
|  | GRIN2B | CHDB | 0.729 |
|  | KATNAL2 | CHD8 | 0.725 |
|  | TBR1 | GRIN2B | 0.717 |
|  | KATNAL2 | SCN2A | 0.713 |
|  | SCN2A | CHD8 | 0.690 |
|  | ARID1B | CHD8 | 0.667 |
|  | MECP2 | NLGN4X | 0.654 |
|  | GRIN2B | SCN2A | 0.610 |
|  | TBR1 | CHD8 | 0.605 |
| **Depression** | HLA-DQB1 | HLA-DQA1 | 0.998 |
|  | KMT2A | EP300 | 0.993 |
|  | HLA-DQB1 | HLA-B | 0.935 |
|  | HLA-DQA1 | HLA-B | 0.929 |
|  | DENND1B | DENND1A | 0.906 |
|  | TCF4 | EP300 | 0.890 |
|  | LACC1 | CCDC122 | 0.846 |
|  | RBFOX1 | CELF4 | 0.673 |
|  | RERE | EP300 | 0.629 |
| **Suicide** | GIMAP5 | GIMAP4 | 0.932 |
|  | GIMAP7 | GIMAP4 | 0.807 |
|  | PFN2 | GSN | 0.794 |
|  | GIMAP1 | GIMAP4 | 0.792 |
|  | GIMAP7 | GIMAP5 | 0.791 |
|  | GIMAP7 | GIMAP1 | 0.711 |
|  | HTR2A | CACNA1D | 0.657 |
|  | RCBTB2 | FNDC3A | 0.655 |
|  | GIMAP5 | GIMAP1 | 0.646 |

**Supplementary Table 3 Correlation coefficients between different psychiatric disorder genes**

| **Gene 1** | **Gene 2** | **Correlation score** |
| --- | --- | --- |
| CACNB2 | CACNA2D1 | 0.995 |
| CACNA2D1 | CACNA1C | 0.983 |
| CACNB2 | CACNA1C | 0.967 |
| DLGAP1 | NLGN4X | 0.953 |
| GRM8 | GRM3 | 0.911 |
| TBR1 | PAX6 | 0.907 |
| CACNA1C | CACNA1E | 0.763 |
| GRIA1 | DLGAP1 | 0.725 |
| GRIA1 | DRD2 | 0.716 |
| ANK2 | CACNA1C | 0.711 |
| GRIA1 | GRIK2 | 0.669 |
| GRM8 | GRIA1 | 0.662 |
| TBR1 | LHX2 | 0.657 |
| NRG1 | ZNF804A | 0.631 |
| CACNA1I | CACNA1E | 0.600 |

**Supplementary Table 4a Correlation coefficient between high expression of psychiatric disorder-related genes in different cancer types**

| **Cancer type** | **Gene 1** | **Gene 2** | **Correlation score** |
| --- | --- | --- | --- |
| ACC | TBR1 | LHX2 | 0.657 |
|  | NR4A2 | LHX2 | 0.615 |
| KIRC | ATP1A3 | ABCB8 | 0.737 |
|  | TBR1 | POU3F2 | 0.692 |
|  | TBR1 | LHX2 | 0.657 |
| LGG | HLA-DQB1 | HLA-DQA1 | 0.998 |
|  | HLA-DQB1 | HLA-B | 0.935 |
|  | HLA-DQA1 | HLA-B | 0.929 |
|  | STAT1 | ERBB2 | 0.910 |
| UVM | HLA-DQB1 | HLA-DQA1 | 0.998 |
|  | GNAS | ADCY2 | 0.978 |
|  | DRD2 | ADCY2 | 0.956 |
|  | GRM8 | DRD2 | 0.940 |
|  | HLA-DQB1 | HLA-B | 0.935 |
|  | HLA-DQA1 | HLA-B | 0.929 |
|  | GRM8 | ADCY2 | 0.907 |
|  | GIMAP4 | GIMAP1 | 0.792 |
|  | GRM8 | GNAS | 0.743 |

**Supplementary Table 4b Correlation coefficient between low expression of psychiatric disorder-related genes in different cancer types**

| **Cancer type** | **Gene 1** | **Gene 2** | **Correlation score** |
| --- | --- | --- | --- |
| KIRC | KMT2A | EP300 | 0.993 |
|  | NRG1 | ERBB2 | 0.992 |
|  | SHANK2 | NLGN4X | 0.976 |
|  | HDAC5 | EP300 | 0.962 |
|  | RORA | EP300 | 0.958 |
|  | SHANK2 | GRM5 | 0.957 |
|  | NLGN4X | GRM5 | 0.950 |
|  | KMT2A | CHD8 | 0.946 |
|  | SYNE2 | SYNE1 | 0.928 |
|  | TCF4 | EP300 | 0.890 |
|  | CHD8 | EP300 | 0.718 |
|  | SYNE1 | ANK3 | 0.688 |
|  | DGKH | ANK3 | 0.669 |
|  | CHD8 | ARID1B | 0.667 |
|  | RCBTB2 | FNDC3A | 0.655 |
|  | NLGN4X | MECP2 | 0.654 |
|  | RERE | EP300 | 0.629 |
|  | MECP2 | EP300 | 0.610 |
|  | GPD2 | DGKH | 0.609 |
| LGG | SHANK2 | DLGAP1 | 0.994 |
|  | SHANK2 | NLGN1 | 0.969 |
|  | GRIN2B | GRIA1 | 0.955 |
|  | PC | LDHB | 0.952 |
|  | NLGN1 | DLGAP1 | 0.952 |
|  | FADS2 | ACSL6 | 0.922 |
|  | PTGFR | HTR2A | 0.905 |
|  | NLGN1 | GRIN2B | 0.818 |
|  | GRIN2B | DLGAP1 | 0.769 |
|  | CACNA1E | CACNA1C | 0.763 |
|  | GRID2 | GRIA1 | 0.740 |
|  | NEGR1 | FAIM2 | 0.737 |
|  | CACNA1C | HTR2A | 0.730 |
|  | DLGAP1 | GRIA1 | 0.729 |
|  | NLGN1 | GRIA1 | 0.722 |
|  | ZNF804A | CACNA1C | 0.709 |
|  | GRIN2B | GRID2 | 0.699 |
|  | GRIK2 | GRIA1 | 0.669 |
|  | SHANK2 | GRIA1 | 0.633 |
|  | SHANK2 | GRIN2B | 0.632 |
|  | GRIK2 | GRID2 | 0.631 |
|  | GRIN2B | GRIK2 | 0.626 |
|  | RBFOX1 | CELF4 | 0.673 |
|  | PAX6 | LHX2 | 0.670 |
|  | PAX6 | ELP4 | 0.633 |
|  | RERE | EP300 | 0.629 |
|  | NR4A2 | LHX2 | 0.615 |
|  | DRD2 | HTT | 0.604 |

**Figure 5a Functional enrichment analysis of high expression genes of different psychiatry disorders**

| **Psychiatric disorder** | **Genes** | **Reactome pathways** | **Molecular Function** |
| --- | --- | --- | --- |
| **Bipolar disorder** | VRK2 | - | MAP-kinase scaffold activity; mitogen-activated protein kinase kinase binding |
|  | RHEBL1 | PIP3 activates AKT signaling | GTPase activating protein binding; Hsp90 protein binding |
|  | CACNA1C | Presynaptic depolarization and calcium channel opening; Muscle contraction | N-terminal myristoylation domain binding; adenylate cyclase activator activity; protein phosphatase activator activity; high voltage-gated calcium channel activity; inositol-1,4,5-trisphosphate 3-kinase activity |
|  | DDN | Neurotransmitter receptors and postsynaptic signal transmission | glutamate receptor binding |
|  | FADS2 | Metabolism | decanoate-CoA ligase activity; long-chain fatty acid-CoA ligase activity; fatty acid ligase activity; very long-chain fatty acid-CoA ligase activity; CoA-ligase activity |
|  | PC | The citric acid (TCA) cycle and respiratory electron transport; Metabolism | pyruvate kinase activity; phosphoenolpyruvate carboxykinase (GTP) activity; malate dehydrogenase (decarboxylating) (NADP+) activity; malate dehydrogenase (decarboxylating) (NAD+) activity; oxaloacetate decarboxylase activity |
| **Anxiety disorder** | THBS2 | Degradation of the extracellular matrix; Disease; Hemostasis | fibronectin binding; laminin binding;  extracellular matrix binding; coreceptor activity; collagen binding |
| **Schizophrenia** | DRD2 | Opioid Signalling; Signal Transduction | group II metabotropic glutamate receptor activity; opioid peptide activity; opioid receptor binding; dopamine binding;  neuropeptide hormone activity |
|  | CACNA1C | Same as above | Same as above |
| **OCD** | CASC8 | - | - |
|  | CASC11 | - | - |
|  | PTPRD | Neuronal System; Neurotransmitter release cycle | - |
| **PTSD** | TLL1 | Extracellular matrix organization | Extracellular matrix organization |
|  | PRTFDC1 | Metabolism | purine phosphoribosyltransferase activity; transferase activity, transferring pentosyl groups; ligase activity, forming carbon-nitrogen bonds; magnesium ion binding; transferase activity, transferring one-carbon groups |
| **Major depression disorder** | VRK2 | Same as above | Same as above |
|  | MLF1 | Amplification of signal from the kinetochores | - |
|  | TENM2 | - | - |
|  | DRD2 | Same as above | Same as above |
|  | HLA-B | ER-Phagosome pathway; Adaptive Immune System | HLA-B specific inhibitory MHC class I receptor activity; TAP binding; peptide antigen binding;  T cell receptor binding; beta-2-microglobulin binding |
|  | XRCC3 | Reproduction; HDR through Homologous Recombination (HRR) | telomeric G-quadruplex DNA binding; forked DNA-dependent helicase activity; 8-hydroxy-2'-deoxyguanosine DNA binding; telomeric D-loop binding; recombinase activity |
|  | BCHE | - | apolipoprotein binding; pyridoxal phosphate binding; chaperone binding; antioxidant activity;  coenzyme binding |
|  | EPHB2 | Developmental Biology | transmembrane-ephrin receptor activity; ephrin receptor binding; protein tyrosine kinase activity; virus receptor activity; Rho guanyl-nucleotide exchange factor activity |
|  | ATP1A3 | Transport of small molecules; Hemostasis | sodium:potassium-exchanging ATPase activity; sodium channel regulator activity; ATPase activator activity; ATPase binding; ATPase activity, coupled to movement of substances |
| **Suicide** | PREX1 | Hemostasis; Signal Transduction | Rho GDP-dissociation inhibitor binding; thioesterase binding; GTPase activity; GDP binding;  GTP binding |
|  | LACTB | RNA polymerase II transcribes snRNA genes; Adaptive Immune System | metalloaminopeptidase activity; peptide binding;  peptidase activity; hydrolase activity; catalytic activity, acting on a protein |
|  | SP140 | - | adenylyltransferase activity |
|  | ABCB8 | Mitochondrial ABC transporters; ABC transporters in lipid homeostasis; Transport of small molecules | sodium:potassium-exchanging ATPase activity; ATPase-coupled transmembrane transporter activity; ATPase activity, coupled to movement of substances; lipid transporter activity; ATP binding |
|  | SLC7A1 | Amino acid transport across the plasma membrane; Neurotransmitter release cycle | L-glutamine transmembrane transporter activity;  high-affinity glutamate transmembrane transporter activity; sulfur amino acid transmembrane transporter activity; L-serine transmembrane transporter activity; neutral amino acid transmembrane transporter activity |
|  | GIMAP4 | - | GTP binding; serine-type endopeptidase activity; protein homodimerization activity; peptidase activity; identical protein binding |
|  | CRISPLD2 | - | - |
|  | GNAS | Opioid Signalling; Hemostasis; Signal Transduction | adenylate cyclase binding; adenylate cyclase activity; guanylate cyclase activity; peptide hormone receptor binding; spectrin binding |
|  | PFN2 | Developmental Biology; Hemostasis | profilin binding; actin monomer binding; SH3 domain binding; structural constituent of cytoskeleton; actin binding |

**Figure 5b Functional enrichment analysis of low expression genes of different psychiatry disorders**

| **Psychiatric disorder** | **Genes** | **Reactome pathways** | **Molecular Function** |
| --- | --- | --- | --- |
| **Bipolar disorder** | TMEM108 | - | integrin binding; motor activity; microtubule binding; protein C-terminus binding; protein kinase binding |
|  | ADCY2 | Opioid Signalling; Hemostasis; Signal Transduction | dopamine neurotransmitter receptor activity, coupled via Gs; cAMP-dependent protein kinase regulator activity; cAMP-dependent protein kinase activity; dopamine neurotransmitter receptor activity; dopamine binding |
|  | ITIH1 | - | immunoglobulin binding; serine-type endopeptidase inhibitor activity; heme binding; cofactor binding; enzyme regulator activity |
|  | HDAC5 | PIP3 activates AKT signaling; Developmental Biology | NAD-dependent histone deacetylase activity (H3-K14 specific); activating transcription factor binding; histone deacetylase binding; RNA polymerase II activating transcription factor binding; protein deacetylase activity |
| **Anxiety disorder** | TMEM132D | - | fatty acid binding; carboxylic acid binding; lipid binding |
|  | FGD2 | Hemostasis; Signal Transduction | thioesterase binding; GTPase activity; GTP binding; Rho GTPase binding; kinase regulator activity |
|  | PDE4B | Opioid Signalling; Hemostasis; Metabolism | cAMP-dependent protein kinase activity; nucleoside kinase activity; nucleobase-containing compound kinase activity; magnesium ion binding; kinase activity |
| **Autism** | KATNAL2 | - | modification-dependent protein binding; helicase activity; catalytic activity, acting on DNA; ATPase activity; ATP binding |
| **PTSD** | ANKRD55 | Organelle biogenesis and maintenance | - |
| **Major depression disorder** | SLC30A9 | Insulin processing; Transport of small molecules | zinc ion transmembrane transporter activity; metal ion transmembrane transporter activity; cation transmembrane transporter activity |
|  | LRP1B | - | frizzled binding; cell adhesion molecule binding |
|  | FHIT | Apoptosis | bis(5'-adenosyl)-triphosphatase activity; SUMO transferase activity; disordered domain specific binding; p53 binding; protein N-terminus binding |
|  | HTT | Membrane Trafficking | - |
|  | ATP1A3 | Transport of small molecules; Hemostasis | sodium:potassium-exchanging ATPase activity; sodium channel regulator activity; ATPase activator activity; ATPase binding; ATPase activity, coupled to movement of substances |
|  | SERPING1 | Complement cascade; Hemostasis | serine-type endopeptidase activity; serine-type endopeptidase inhibitor activity; endopeptidase inhibitor activity; calcium ion binding |
| **Suicide** | ABI3BP | Extracellular matrix organization | heparin binding; growth factor binding |
|  | PDE4B | Same as above | Same as above |
|  | MRAP2 | Signal Transduction | melanocyte-stimulating hormone receptor activity; melanocortin receptor activity; neuropeptide binding; peptide hormone binding; hormone binding |
|  | ACSL6 | Metabolism | palmitoyl-CoA oxidase activity; acyl-CoA oxidase activity; decanoate-CoA ligase activity; acyl-CoA dehydrogenase activity; long-chain fatty acid-CoA ligase activity |
|  | GIMAP1 | - | GTP binding; ion binding; heterocyclic compound binding; organic cyclic compound binding |
|  | GIMAP7 | - | GTP binding; purine ribonucleoside triphosphate binding; purine ribonucleotide binding |
|  | GIMAP4 | - | GTP binding; serine-type endopeptidase activity; protein homodimerization activity; peptidase activity; identical protein binding |
|  | GIMAP5 | - | GTP binding; protein homodimerization activity |
|  | MSRA | Metabolism of proteins | L-methionine-(R)-S-oxide reductase activity; peptide-methionine (R)-S-oxide reductase activity; oxidoreductase activity, acting on a sulfur group of donors, disulfide as acceptor; transferase activity, transferring alkyl or aryl (other than methyl) groups |
|  | CACNA1D | Presynaptic depolarization and calcium channel opening; Muscle contraction | high voltage-gated calcium channel activity; voltage-gated calcium channel activity; calcium channel activity; voltage-gated ion channel activity; ion gated channel activity |
|  | RETREG1 | - | chloride channel inhibitor activity |
|  | SYTL3 | Innate Immune System | - |
